# Cellular and Molecular Effects of Microgravity on the Immune System: A Focus on Bioactive Lipids

**DOI:** 10.3390/biom14040446

**Published:** 2024-04-05

**Authors:** Marina Fava, Noemi De Dominicis, Giulia Forte, Monica Bari, Alessandro Leuti, Mauro Maccarrone

**Affiliations:** 1 Department of Medicine, Campus Bio-Medico University of Rome, Via Alvaro del Portillo 21, 00128 Rome, Italy; m.fava@unicampus.it (M.F.); g.forte@unicampus.it (G.F.);; 2 European Center for Brain Research/IRCCS Santa Lucia Foundation, Via del Fosso di Fiorano 64, 00143 Rome, Italy; 3 Department of Physics, University of Trento, 38123 Trento, Italy; noemi.dedominicis@student.univaq.it; 4 Department of Biotechnological and Applied Clinical Sciences, University of L’Aquila, 67100 L’Aquila, Italy

**Keywords:** microgravity, inflammation, bioactive lipids, endocannabinoids, eicosanoids

## Abstract

Microgravity is one of the main stressors that astronauts are exposed to during space missions. This condition has been linked to many disorders, including those that feature dysfunctional immune homeostasis and inflammatory damage. Over the past 30 years, a significant body of work has been gathered connecting weightlessness—either authentic or simulated—to an inefficient reaction to pathogens, dysfunctional production of cytokines and impaired survival of immune cells. These processes are also orchestrated by a plethora of bioactive lipids, produced by virtually all cells involved in immune events, which control the induction, magnitude, outcome, compartmentalization and trafficking of immunocytes during the response to injury. Despite their crucial importance in inflammation and its modulation, however, data concerning the role of bioactive lipids in microgravity-induced immune dysfunctions are surprisingly scarce, both in quantity and in variety, and the vast majority of it focuses on two lipid classes, namely eicosanoids and endocannabinoids. The present review aims to outline the accumulated knowledge addressing the effects elicited by microgravity—both simulated and authentic—on the metabolism and signaling of these two prominent lipid groups in the context of immune and inflammatory homeostasis.

## 1. Introduction

Space missions conducted in the last decades have helped clarifying the ability of human physiology to adapt to stressors that cannot be found on Earth, with microgravity being undoubtedly the one that has catalyzed the greatest attention in recent years.

In particular, the role of microgravity in space-related alterations was initially hypothesized following the observation that more than half of the astronauts in the Apollo missions displayed enhanced susceptibility to microbial infections [1]. In the following decades, Augusto Cogoli’s pioneering studies revealed that increased infections were likely to be caused by suppression and alteration of lymphocyte functions elicited by the time spent in microgravity [2,3,4].

Since then, a multitude of other works reported that microgravity—either authentic or simulated—can alter cell size, viability, morphology, polarity, functionality, proliferation and signaling, and demonstrated that exposure to weightlessness can induce a number of disorders, including altered bone remodeling, muscle atrophy and vascular/cardiovascular dysfunctions [5,6,7,8]. On the other hand, understanding the molecular mechanisms of space-related immune alterations—especially those linked to microgravity—is of the utmost importance for future missions and prolonged travel in space.

In this context, bioactive lipids, such as eicosanoids, endocannabinoids, specialized pro-resolving mediators (SPMs), sphingolipids and various lysophospholipids orchestrate the entire immune response by controlling its initiation, termination and, possibly, deviant behaviors [9].

The initial phase of a phlogistic event is generally characterized by the production of different pro-inflammatory cytokines, as well as by the biosynthesis of arachidonic acid (AA)-derived eicosanoids that drive the cardinal signs of acute inflammation: redness, swelling, heat and pain. Since inflammation is designed, at least in principle, to be a defensive self-limiting system that avoids microbe invasion and dissemination, it is supposed to be confined and shut down after the immune response, in a process that has been called “resolution of inflammation” during which SPMs tame down the immune system and avoid chronic damage [10]. However, dysregulated production of pro-inflammatory mediators, injury or pathogen endurance, or impairment of the resolution network leads to chronic and perpetuated inflammation that triggers fibrosis and loss of tissue function [10].

All bioactive lipids have been involved in inflammatory homeostasis, and exposure to microgravity has been extensively linked to immune-related disorders; yet, studies that addressed the role of endogenous lipids in microgravity-related pathways are few and almost exclusively focused on two main families: eicosanoids and endocannabinoids.

## 2. Effects of Microgravity on Inflammation

Inflammation is a crucial mechanism that serves as the body’s first line of defense against infection and tissue injury of any nature and plays a role in virtually every known pathology [9,11] An acute inflammatory response involves interactions between tissues and immune cells—mostly innate, like neutrophils and monocytes/macrophages [9], and it is designed to assay the injury site in search for pathogens to be promptly eliminated [9,10,12]. Conversely, should the inflammatory response persist—due to either elusive pathogens, deranged immune signaling or impaired resolution—inflammation transitions into a chronic state, in which a vicious cycle of pro-inflammatory mediators (i.e., lipids and cytokines/chemokines) leads to fibrosis and loss of tissue function [9,13]. In general, any condition that leads to a dysregulated immune response can lead to the onset of pathologies.

Relevant to this, microgravity-induced alterations of the immune and inflammatory response represent the main mechanism behind the pathologies associated with space travel. Indeed, it has become clear that during space missions, the human body undergoes different kinds of psychological, physical and biological modifications, which give rise to morphological, functional and biochemical imbalances, many of which result in malfunction of the immune system [14,15].

Many studies have elucidated that the cells of the immune system—in particular lymphocytes—undergo a change in both number and function following exposure to weightlessness, which leads to the establishment of immune dysfunction and prolonged inflammation over time [16,17,18].

The earliest reports about disturbed immune cell functions in space date back to 1975, when increased viral or bacterial infections due to the suppression of lymphocyte function were discovered in crew members of Soyuz, Skylab and Apollo missions, inflight or immediately after landing [1]. 

This discovery has been corroborated by several independent studies, where it was reported that authentic microgravity, or simulated weightlessness in a random positioning machine (RPM) or two-dimensional clinostat, can affect T lymphocyte proliferation and cytokine production in response to various T cell receptor (TCR) agonists, such as concanavalin A (ConA) and anti-CD3/CD28 antibodies [19,20,21]. Similarly, peripheral blood mononuclear cells (PBMCs) isolated from astronauts, which were collected and stimulated just after landing on Earth, showed a significant reduction in CD4+ T cell-derived interferon (INF)-γ [22]. Even though the mechanism underlying this reduced activation might be quite intricate, some authors have suggested that low proliferation and inhibition of cytokine production in the presence of mitogens was due to lower cell-to-cell contact. In line with this, the mitogen-induced proliferation of T lymphocytes is known to depend on their interaction with accessory cells and is mediated by additional signals including soluble substances such as IL-2; of note, the production of this cytokine may decrease due to the pressure changes that cells experience in weightlessness [23]. In this context, a parabolic flight study by Tauber and colleagues showed that cell surface expression of CD3 and of the IL-2 receptor CD25 on non-activated human T cells decreased 20 s after simulated microgravity, suggesting that alteration of immune signals can occur quite early in the absence of gravity [24]. Similar studies have confirmed an alteration of the mitogen-driven surface signals in lymphocytes, as shown by the fact that T cells undergoing weightlessness display a downregulated CD25 [3,25]. 

It should be noted that, although most studies agree on the type of alterations microgravity can elicit on immune response, there is not yet a clear consensus on the effects that might depend on the time of exposure to this stressor. For instance, a recent study has shown that splenic mouse T lymphocytes exposed for different periods of time to simulated weightlessness display different responses to ConA [26]:in particular, T cells underwent a significant time-dependent downregulation of several markers, such as CD25, CD69 and pro-inflammatory cytokines like IL-2 and IFNγ. This same study also highlighted a differential susceptibility of T cell subsets to simulated microgravity, with CD4+ showing decreased proliferation and cytokine production compared to CD8+ [26].

It is remarkable that some alterations occur only after short and acute exposures to microgravity but are recovered over longer periods, as suggested by investigations showing that reduced INF-γ production in T cells is associated with short-time flights (i.e., on the Space Shuttle) while being absent in long-time missions (i.e., on board the ISS) [27,28].

Spaceflight can also act on the inflammatory response by targeting innate immune cells. A seminal study conducted nearly four decades ago reported altered numbers of polymorphonuclear cells (PMNs) in astronauts who underwent spaceflight on board the Space Shuttle, with increased neutrophils and decreased eosinophils [29]. This evidence might suggest some acute stress induced by spaceflight on astronauts’ innate immune response. In line with this, a more recent study using 30 consecutive parabolic flights (each featuring 20 s of microgravity) showed decreased blood levels of soluble factors associated with innate immune activation (e.g., platelet-derived growth factor AA and BB and eotaxin) [30].

Quite surprisingly, monocytes have received little attention in microgravity studies, despite their pivotal role as initiators and orchestrators of the immune response. The few studies published so far showed reduced expression of CD62L and HLA, decreased production of IL-6, TNF-α and IL-10 following LPS stimulation [31], diminished phagocytosis, oxidative burst and degranulation [18], as well as a significant cytoskeletal alteration, which in turn influenced their motility [32].

Accordingly, microgravity also impairs macrophage development from hematopoietic stem cells by altering the Ras/Erk/NF-κB pathway [33], while eliciting metabolic reprogramming and altering cytokine production [34]. Interestingly, also complement proteins–which are known affect macrophage activation and chemotaxis–have been suggested as a target of microgravity [35].

Studies that interrogated the effect of microgravity on natural killer (NK) cells have generally reported that authentic or simulated weightlessness reduces the activation or circulating numbers of these cells. Studies conducted in the early 1980s showed decreased activity and INF-γ production in the NK cells of astronauts who underwent a 7-day spaceflight [36], while more recent works reported that these cells were the only lymphoid population being reduced after 520 days of simulated space travel in the context of the Mars-500 mission [37]. Yet, other authors have shown that NK cells cultured in simulated microgravity produce less IFN-γ and perforin, although cytotoxicity was recovered quickly within the first 3 days of culture at Earth (1× *g*) gravity and recovered completely between 3 and 5 days. On the other hand, a single study failed to show changes in either the number or cytotoxicity of NK cells in astronauts who were on board the ISS or exposed to simulated weightlessness in ground studies [23].

Finally, recent studies have also reported a direct effect of microgravity on endothelial cells (ECs), which possess essential secretory, synthetic, metabolic and immunologic activities. After exposure to microgravity, ECs showed an increase in apoptosis [38,39], a downregulation of adhesion molecules that drive immunocyte recruitment [40], and enhancement of the Nlrp3-dependent inflammatory cascade [41].

## 3. Bioactive Lipids and Their Involvement in Microgravity

Endogenous lipids are thought to act together as they orchestrate and shape immune response and tissue homeostasis. Nevertheless, to date, only a few studies have addressed alterations in bioactive lipid signaling in either authentic or simulated microgravity, mostly focusing on AA-derived eicosanoids and endocannabinoids. Furthermore, although several groups have recently investigated the molecular mechanisms behind spaceflight-induced immune alterations, the full array of molecular actors behind these conditions remains mostly elusive, with most of the available literature data covering cytokine biology.

Understanding the engagement of lipid signals in the modifications triggered by microgravity on human immune networks represents a crucial piece of the puzzle that will ultimately enable safe space travel in the near future. Generally speaking, endogenous lipids are molecules characterized by hydrophobic or amphiphilic structures that are biologically relevant not only for energy supply but also for cell membrane architecture and cell signaling [12], with eicosanoids and endocannabinoids exerting very diverse functions such as control of the inflammatory balance, immune modulation, release of synaptic neurotransmitters, gastric secretion and blood clotting [42,43].

### 3.1. Metabolism and Signaling of Eicosanoids, and Their Involvement in Immune Response

Classical eicosanoids are the lipid family that has been better characterized, to date, and includes several molecules sharing arachidonic acid (AA) as their common biosynthetic precursor [12].

The synthesis of eicosanoids begins upon the release of the membrane-tethered AA, an ω-6 polyunsaturated fatty acid (PUFA), via the action of the cytosolic phospholipase A_2_ (cPLA_2_), a Ca^2+^-dependent esterase that cleaves the *sn-2*-bound fatty acid of phospholipids [44]. The AA is then transformed into the final bioactive products via three main pathways driven by: (i) cyclooxygenases (COXs), (ii) lipoxygenases (LOXs) and (iii) cytochrome P450 epoxygenases (CYP450) [9].

(i) Cyclooxygenase 1 and 2 (COX-1 and COX-2) oxidize AA to prostaglandin G_2_ (PGG_2_) and then to prostaglandin H_2_ (PGH_2_), which is then processed into other prostaglandins (i.e., PGI_2_, PGE_2_, PGF_2α_ and PGD_2_) and thromboxane A_2_ (TXA_2_) by the action of selective synthases [45,46].

Each COX-derived lipid engages and activates a specific G protein-coupled receptor (GPCR) (or a group thereof), with PGD_2_ acting on DP1 and DP2 receptors, PGE_2_ engaging EP class receptors (i.e., EP1-4), and PGF_2α_, PGI_2_ (often referred to as prostacyclin) and TXA_2_ binding FP, IP and TP receptors, respectively [43].

(ii) Lipoxygenase (LOX) isozymes convert AA into non-cyclic molecules involved in inflammation, namely leukotrienes (LTs), lipoxins (LXs) and hepoxilins (HXs); in particular, AA is converted into 5-, 12- and 15-hydroperoxyeicosatetraenoic acid (5-, 12-, 15-HpETE) by the stereospecific action of 5-, 12- and 15-LOX, respectively [9]. 5-LOX is also responsible for the conversion of 5-HpETE into LTA_4_, the common precursor to all LTs: cysteinyl-LTs (CysLT, i.e., LTC_4_, LTD_4_ and LTE_4_) are generated from LTC_4_ by LTC_4_ synthase (LTC_4_S), an enzyme with glutathione *S*-transferase activity, while the non-CysLTs LTB_4_ does present glutathione in its backbone and is produced through the hydrolysis of the LTC_4_ epoxide by LTC_4_ hydrolase (LTC_4_H) [43]. CysLT receptors 1 and 2 (CysLTR1-2) and their ligands are profoundly involved in pulmonary inflammation, as well as being the main targets of antiasthmatic drugs such as montelukast, zafirlukast and pranlukast, which act as their selective antagonists [47]. Moreover, LTB_4_ receptors (i.e., BLT_1-2_) are part of an intricate network that involves both inflammation and resolution, with resolvin E1 (RvE1, an eicosapentaenoic acid (EPA)-derived SPM) acting as a partial agonist/antagonist to BLT_1_ [48]. Finally, 12-LOX-dependent conversion of AA into 12-HpETE kickstarts the synthesis of a small group of pro-inflammatory compounds called hepoxilin A and B (HXA and HXB), which can engage transient receptor potential ankyrin 1 and vanilloid 1 (TRPA1 and TRPV1) channels [43].

(iii) Cytochrome P450 epoxygenases (CYP450) convert AA into a series of epoxyeicosatetraenoic acids (EETs) that can engage peroxisome proliferator-activated receptors α and γ (PPARα and PPARγ). The latter are a class of receptors deeply involved in the signal transduction of EETs as well as of many other lipid congeners, including PGs, LOX-derived intermediates of AA and endocannabinoids [43,49].

Prostaglandins and thromboxanes are conventionally linked to acute inflammation establishment, while lipoxins are better known for their role in its resolution [10,43,50].

Classical eicosanoids have long been known for their pleiotropic role in immune regulation and inflammatory response, which includes chemotaxis, recruitment, maturation, activation and production of cytokines (e.g., IL10 and TNF) in leukocytes, as well as fever, pain, vascular permeabilityand platelet aggregation [43]. The COX- and LOX-derived compounds possess great scientific, social and historical relevance: indeed, COX-1/COX-2 inhibitors such as aspirin (2-acetoxybenzoic acid or acetylsalicylic acid) and other non-steroidal anti-inflammatory drugs (NSAID) represent both the most prescribed drugs worldwide as well as the oldest known strategy to treat inflammation and pain. Seemingly, salicylate-based preparations were already known to Egyptians and Sumerians [51].

**Figure 1:**
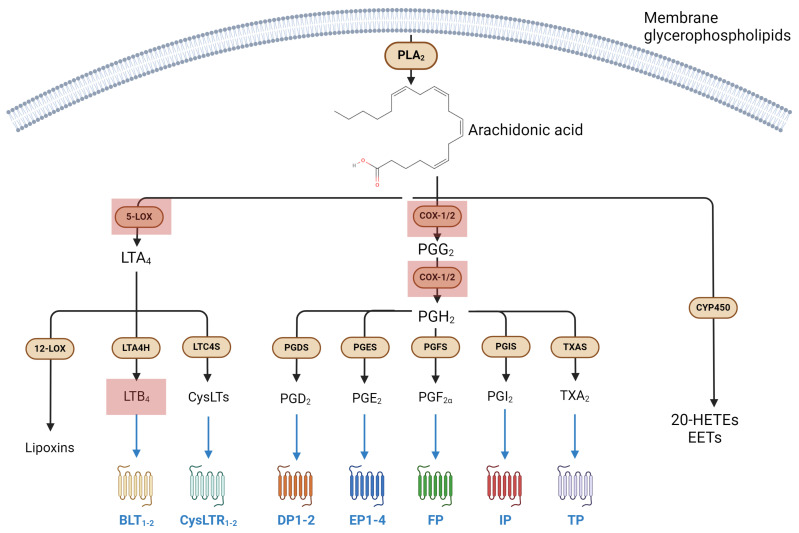
Eicosanoid metabolic pathways and their main receptors. Red squares represent molecular targets affected by microgravity. BLT: leukotriene B_4_ receptor; COX: cyclooxygenase; CYP450: cytochrome P 450; CysLT: cysteinyl leukotriene; DP: PGD_2_ receptor; EP: PGE_2_ receptor; EET: epoxyeicosatetraenoic acid; FP: PGF_2α_ receptor; HETE: 5-hydroxyeicosatetraenoic acid; IP: PGI_2_ receptor; LOX: lipoxygenase; LT: leukotriene; LTA_4_H: LTA_4_ hydrolase; LTC_4_S: LTC_4_ synthase; LXB_4_: lipoxin B_4_; PLA_2_: phospholipase A_2_.

Eicosanoid metabolism and receptor targets are summarized in Figure 1.

#### Eicosanoids in Microgravity

Despite the huge amount of literature accumulated in the past 40 years on eicosanoids as mediators of inflammation, not many studies have investigated their involvement in short or long exposure to microgravity, with most of the available publications having focused on the metabolic enzymes only.

One of the earliest reports on space-related changes in LOXs catalytic properties was reported by our group in the 2000s during the ESA 28th parabolic flight campaign. This study took advantage of the EMEC (Effect of Microgravity on Enzymatic Catalysis) module fiber optics spectrometer to demonstrate that LOX-1 can function as a “gravity sensor”, as suggested by the finding that microgravity increases the catalytic efficiency of this enzyme by increasing its substrate affinity (i.e., by reducing Michaelis–Menten constant, K_m_) without affecting its maximum velocity, V_max_ [52]. LOX-1 is the main lipoxygenase in plants, although it shares several structural and mechanistic features with human lipoxygenases; this gave rise to the idea that other human-derived LOXs could also be affected by microgravity. Accordingly, we were able to demonstrate that 5-LOX displays reduced activity in K562 cells that underwent 12 h of clinorotation-simulated microgravity. Conversely, hypergravity (obtained by incubating cells in a centrifuge at 22× *g*) resulted in a 250% increase in activity with respect to 1× *g* controls [53]. A follow-up to the same study showed a similar result for COX-2 enzyme, which doubled its activity when K562 cells were exposed to hypergravity and decreased it when subjected to microgravity, further supporting the observation that eicosanoid metabolic enzymes do indeed react to gravitational changes. Remarkably, in an independent study that we conducted almost 20 years later on Jurkat cells, which were incubated in the Rotary Cell Culture System (RCCS) developed by NASA to simulate microgravity in a very controlled manner, we found an early activation of 5-LOX and consistently increased LTB_4_ synthesis. Such an enhancement in LTB_4_ levels was also paralleled by increased apoptosis and reduced release of anti-apoptotic cytokines such as LIF, IL-2 and IL-4, as well as by a significant increase in the production of pro-apoptotic cytokine INF-γ [54]. These data corroborate the role of LOX enzymes—and of their metabolites—as key players in the adaptation to microgravity and might explain alterations in the immune response upon exposure to weightlessness. In particular, LTB_4_ acts as a powerful chemoattractant for inflammatory cells and induces degranulation, superoxide anion production and adherence of neutrophils to vascular endothelial cells of inflamed sites [55]; thus, variations in its biosynthetic tone in microgravity might hold great importance in explaining the cellular processes of space-related immune alterations. We were also able to corroborate these studies by using authentic microgravity on human PBMCs on board the ISS in the frame of the ROALD mission [56]. During this experiment, not only could we confirmed that human PBMCs display enhanced apoptosis-associated markers, but also that this feature was due to increased 5-LOX activity [56].

It should be noted that COXs and LOXs represent the two main enzymes controlling eicosanoid levels during inflammation; therefore, these data suggest that microgravity might have an impact on phlogistic processes by targeting AA-derived endogenous lipids. In addition, LOXs are well-known modulators of immune cell biology [12], and their alteration during spaceflight might represent one of the causes leading to the microgravity-related impairment of lymphocyte function. Notably, few works have addressed prostaglandin levels—or expression of prostaglandin-related elements—in gravity-related studies in recent times, and none of them directly addressed immunity. In particular, prostaglandin transporter gene SLCO2A1 was shown to undergo significant downregulation in HUVEC and EA.hy926 cells exposed to clinostat-simulated microgravity [57], while PGE_2_ has been suggested as an important player in the molecular mechanisms behind microgravity-induced modifications. Indeed, Guo and colleagues have recently reported that PGE_2_ is secreted by osteoblasts under mechanical loading, or in conditions of low bone density, and controls bone density and fat metabolism by triggering hypothalamic expression of tyrosine hydroxylase (TH) and of neuropeptide Y (NPY) through the EP4-expressing sensory nerves of ascending interoceptive pathways [58,59,60]. With regard to this, the authors have shown that hindlimb suspension leads to PGE2/EP4-mediated signaling in sensory interoceptive pathways, which leads to bone loss and adipose tissue lipolysis [60]

Apparently, microgravity also acts by altering membrane balance and ion permeability through the function of Ca^2+^ channels [61,62]. Studies on bone marrow-derived mast cells have documented that simulated microgravity induces a decline in degranulation and pro-inflammatory cytokines, which correlates with decreased Ca^2+^ in responses to ionomycin. Thus, despite the little information yet available on this subject, it seems fair to anticipate that microgravity contributes to immune dysfunctions through altered Ca^2+^ regulation and Ca^2+^-related functions. In keeping with this view, Ca^2+^ ions play a central role in several cell functions such as motility, chemotaxis and, notably, AA-related synthesis of leukotrienes and prostaglandins [63].

### 3.2. Metabolism and Signaling of Endocannabinoids, and Their Involvement in Immune Response

Endocannabinoids (eCBs) are bioactive lipids ubiquitously and endogenously produced by all cells in the body. *N*-Arachidonoylethanolamine (anandamide, AEA) and 2-arachidonoylglycerol (2-AG) are the two most prominent compounds of this group, and along with their target receptors and metabolic enzymes, constitute the “eCB system (ECS)” [9].

AEA biosynthesis starts with the *N*-acylation of membrane phosphatidyl-ethanolamine, catalyzed by *N*-acyltransferase (NAT) to yield *N*-arachidonoyl-phosphatidylethanolamine (NArPE), which is then hydrolyzed to AEA by the *N*-acyl-phosphatidylethanolamines-specific phospholipase D (NAPE-PLD). On the other hand, most 2-AG is synthesized through the action of diacylglycerol lipases α and β (DAGLα/β) on membrane-derived diacylglycerol (DAG), which is produced by the action of phosphohydrolases on membrane glycerophospholipids [64]. A collateral biosynthetic pathway of 2-AG requires PLA_1_-dependent cleavage of membrane phosphatidylinositol into 2-AG-3-phosphate (2-AG-3P), which isthen hydrolyzed to 2-AG by the action of PLC [9,64].

On the other hand, AEA and 2-AG degradation is led by the fatty acid amide hydrolase (FAAH) and monoacylglycerol lipase (MAGL) enzymes, respectively [65]. In the former case, AEA is degraded into AA and ethanolamine, whereas in the latter, 2-AG is cleaved into AA and glycerol [64]. Furthermore, eCBs can be metabolized via oxidation operated by COXs and LOXs, much like AA [9,65].

AEA and 2-AG exert their biological actions by binding to and activating cannabinoid 1 and 2 (CB_1_ and CB_2_) receptors, two GPCRs associated with heterotrimeric G_i/o_ proteins [66]. Interestingly, CB_1_ receptors are widely distributed in the central nervous system (i.e., brain cortex, limbic system, substantia nigra and hippocampus), whereas CB_2_ receptors are mainly found in immune cells—where it exerts immunosuppressive actions—but also in astrocytes and microglia [67]. Moreover, CB_2_ appears predominant under physiological conditions and upon acute and chronic inflammation, whereas CB_1_ mainly exerts a neuromodulatory and immunoregulatory role [65].

In addition to CB_1_/CB_2_ receptors, eCBs may exert their biological activity by binding to non-canonical receptors, including the transient receptor potential vanilloid type-1 (TRPV1) ion channel, the nuclear PPARs and the orphan G protein-coupled receptors GPR55 and GPR119 [9].

Over the past two decades, these signaling lipids, initially described as neuromodulators/neurotransmitters, have emerged more and more as critical mediators of many aspects of human pathophysiology, showing a remarkable complexity in their mechanisms of biosynthesis, transport, and degradation, and thus displaying manifold immunomodulatory activities. [65]. It should be noted that, alongside proper eCBs, other chemically-related compounds have been identified in the last two decades that display eCB-like properties; these include *N*-acylethanolamines such as *N*-palmitoylethanolamine, *N*-oleoylethanolamine and *N*-stearoylethanolamine, as well as other less-investigated compounds such as *N*-eicosatrienoylethanolamine and *N*-docosatrienoylethanolamine, sometimes referred to as “autacoid local injury antagonist amides” (or ALIAmides). These molecules exert immunomodulatory properties that are similar to authentic eCBs, yet they do not display a comparable affinity for either CB_1_ or CB_2_ [68,69].

The eCBs exert potent effects on the inflammatory functions governed by both the innate and adaptive branches of the immune system; in particular, as summarized in ref. [70], they act on monocytes/macrophages by inducing phagocytosis, the production of immunomodulatory cytokines (e.g., IL-10) and the expression of anti-inflammatory and neuroprotective markers (e.g., CD200R), while at the same time they inhibit the production of acute pro-inflammatory cytokines such as TNF-α, IL-6, IL-12 and IL-23 [64]. Additional actions on the innate repertoire of immunity include hindering neutrophil and PMN migration and recruitment, and degranulation and maturation of mastocytes [70]. Concomitantly, AEA is widely known to act on T cells by promoting their apoptosis (or blocking their proliferation) and by hindering the secretion of pro-inflammatory or activating cytokines such as IL-2, TNF-α and IFN-γ [70].

**Figure 2:**
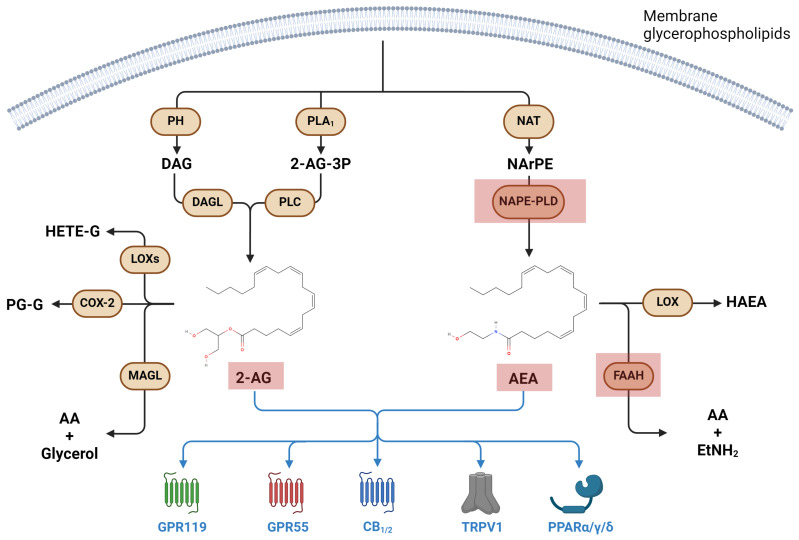
Endocannabinoid metabolic pathways and their main receptors. Red squares represent molecular targets affected by microgravity. 2-AG-3P: 2-arachidonoylglycerol-3-phosphate; 2AG: 2-arachinonoylglycerol; AA: arachidonic acid; AEA: *N*-arachidonoyl ethanolamine; CB: cannabinoid receptor; DAG: diacylglycerol; DAGL: diacylglycerol lipase; EtNH_2_: ethanolamine; FAAH: fatty acid amide hydrolase; GPR: G protein-coupled receptor; HAEA: hydroxyarachidonoylethanolamide; MAGL: monoacylglycerol lipase; NAPE-PLD: *N*-acylphosphatidylethanolamines-specific phospholipase D; NArPE: *N*-arachidonoyl phosphatidylethanolamine; NAT: *N*-acyltransferase; PL: phospholipase; PPAR: peroxisome proliferator-activated receptor; TRPV1: transient receptor potential cation channel subfamily V member 1.

A summary of eCB metabolism and receptor targets is shown in Figure 2.

#### Endocannabinoids in Microgravity

The eCBs have attracted growing interest in space biology, especially because of their vast versatility as immune modulators and their role in the adaptation to many different environmental and metabolic conditions [71,72]. Indeed, imbalances in eCB content, enzyme activity and/or receptor signaling are associated with many inflammatory diseases [9]. Incidentally, it should be noted that eCB metabolism is also regulated by the same LOX isozymes discussed above for AA-derived eicosanoids.

The first report describing a variation in the ECS upon exposure to authentic microgravity originated from the IMMUNO project [71]. This study documented an increase in eCB levels in the blood of crew members, along with an alteration of the immune cell repertoire and functionalities that consisted of: (i) enhanced numbers of neutrophils, NK cells, B cells and monocytes; (ii) reduced numbers of Treg lymphocytes; and iii) overproduction of pro-inflammatory cytokines (e.g., TNF-α and IL-1β) and reduction of anti-inflammatory signals (e.g., TGF-β and IL-10) [71]. It should be noted that the data collected in the first few days of the mission failed to show significant alterations in eCB production or other parameters associated with the ECS. Yet, AEA content was significantly increased in the blood of the astronauts following 150 days post-departure. This study was the first to describe a change in the ECS upon long-time exposure to authentic weightlessness and supported the hypothesis that eCB might be involved in adaptation mechanisms that the body triggers in response to space-related stressors.

In this context, our group significantly contributed to this line of research during the RESLEM experiment, performed on the ISS in 2012 [67], and then during the SERISM experiment, performed in 2017 in the frame of the VITA mission led by ASI (the Italian Space Agency) [73].

The RESLEM data showed that authentic microgravity causes an imbalance in the two enzymes responsible for AEA metabolism in PBMCs; in particular, 48 h of microgravity aboard the ISS caused a time-dependent upregulation of NAPE-PLD and a concomitant downregulation of FAAH, corroborating the idea of AEA as a central molecule in the adaption to microgravity [74].

Of note, 5-LOX also can contribute to controlling AEA production in space because it biosynthesizes hydroperoxide derivatives of AEA, which act as competitive inhibitors of FAAH [56]. As previously described, microgravity induces an early increase in 5-LOX activity in PBMCs, which supports the idea of AEA—and the ECS as a whole—as a main target of microgravity [56]. 

Other studies have also reported an alteration in eCB circulating levels after short (~20 s) parabolic flights [75,76]. AEA and 2-AG levels were measured in the blood of 21 volunteers upon departure and after 10, 20 and 30 parabolas, as well as 24 h after the parabolic flight. Interestingly, AEA blood levels dropped after 10 and 20 parabolas in volunteers who experienced motion sickness (or kinetosis) and high stress scores, while subjects with no symptoms showed amounts of blood AEA that were up to 50% higher than controls. Similarly, 2-AG levels were more abundant in volunteers who did not experience nausea; in particular, these subjects also displayed a lasting increase in 2-AG blood levels, even 24 h after the parabolic flight [76]. These observations suggest that even short periods of weightlessness might cause relevant alterations of the ECS, and that eCB signaling might not only be involved in long-term adaptation to space—where it might impact processes like immune response independently of stress sources—but also as stress-induced mechanisms that are triggered as an adaptive response to immediate environmental modifications.

### 3.3. Additional Bioactive Lipids in Microgravity

The biochemistry of endogenous lipids involved in immune homeostasis encompasses other compounds, such as SPMs, sphingolipids, *lyso*glycerophospholipids or fatty acid esters of hydroxy fatty acids (FAHFAs), which are all involved in inflammatory disorders. However, these classes of lipid signals have been barely addressed in space biology. *Lyso*glycerophospholipids have been recently investigated in a few studies, where both authentic or simulated microgravity caused a significant increase in their production [77,78,79]. In this context, it should also be mentioned that our group recently published the only investigation to date reporting an effect of microgravity on the SPM system, whereby 24 h of RCCS-simulated weightlessness enhanced the expression of SPM receptors GPR32 and GPR18, while reducing the expression of 5-LOX and the level of the prominent SPM resolvin D1 (RvD1) [31]. Similarly, sphingolipids have been scarcely studied. In a recent work conducted on *Caenorhabditis elegans*, spaceflight was shown to induce long-lasting downregulation (i.e., up to twelve days after the experiment) on ceramide-related metabolic pathways, with the main targets being represented by N-acylsphingosine amidohydrolase-1 (ASAH1), acid sphingomyelinase-3 (ASM-3) and glucocerebrosidase-4 (GSB-4) [80]; interestingly, an impairment of sphingolipid metabolism was also confirmed in human erythrocytes cultured in clinorotation, which consisted in a significant downregulation of several sphingomyelins [81], while an alteration of the ASM axis and an impairment of ceramide production was reported in the cerebral and mesenteric artery walls of rats who underwent four weeks of hindlimb suspension [82]. Finally, FAHFAs have only been recently studied in one work, in the context of cosmic radiations, where they were found to be reduced—much like *lyso*glycerophospholipids and sphingolipids– in the serum of mice who underwent long-term brain irradiation with γ rays [83].

## 4. Concluding Remarks

The data collected so far have clearly shown relevant dysfunctions of the immune system during space missions, with recurrent infections and ineffective immune responses, as well as other inflammatory alterations that, however, often revert a few weeks after return to Earth. Available studies strongly suggest a deep alteration of the systems that govern the immune response, both in its cellular and molecular components, of which bioactive lipids represent a crucial backbone.

In this review, we collected both seminal and recent evidence of the effect of microgravity—either authentic or simulated—on lipid systems controlling immune homeostasis. Unfortunately, most of the available data on authentic microgravity are based on pre- and post-flight analyses, because of procedural limitations, or are based on short-term spaceflight missions. Instead, the largest part of research in this field is still conducted on the ground, using instruments that simulate microgravity.

To date, the studies addressing the involvement of bioactive lipids in microgravity and space biology are not only relatively few despite their importance in tissue homeostasis, but they mostly focus on eicosanoids and eCBs, leaving important compounds such as sphingolipids and SPMs—which are crucial in immune regulation—largely, if not completely, unexplored.

These lipid classes have been proven to act both individually and together as a higher-order network and might control adaptation to microgravity. A better understanding of how these signaling networks take part in our response and adaptation to microgravity seems now necessary, to both understand the molecular mechanisms that drive space-related alterations and design countermeasures that make space travel safe. The data reviewed in the present work are summarized in Table 1.

## Figures and Tables

**Table 1:** Effects of microgravity on immune cells and on enzymes and receptors involved in bioactive lipid metabolism.

Cellular/Molecular Target	Effect after Exposure to Microgravity	Method	Reference
Neutrophils	Increased numbers	ISS Space Shuttle flights	[71] [29]
		Parabolic flights (30 parabolas with a duration of 20 s of microgravity)	[30]
Eosinophils	Reduced numbers	Space Shuttle flights	[29]
NK cells	Increased numbers	ISS	[71] [23] [36]
	Reduced INF-γ and perforin production and activity.		
B cells	Increased numbers	ISS	[71]
	Reduced numbers	Parabolic flights (30 parabolas with a duration of 20 s of microgravity)	[30]
T cells	Decreased activation and proliferation	Clinorotation Space Shuttle flights	[22] [3] [25] [19] [27]
		ISS	[23]
		Post flight immune assessment	[28]
		Parabolic flights (30 parabolas with a duration of 20 s of microgravity)	[30]
Monocytes	Increased numbers	ISS	[71] [18] [32]
	Reduced expression of both CD62L and HLA; reduced IL-6, TNFα and IL-10 after LPS stimulation; reduced phagocytosis, oxidative burst and degranulation		
	Cytoskeletal alterations		
Hematopoietic Stem Cells	Alteration of Ras/Erk/NF-κB pathway, alteration of complement system activation and alteration of cytokine production.	RCCS (10^−2^ g) RCCS (10^−2^–10^−3^ g)	[33] [35]
Endothelial cells	Increased apoptosis, down-regulation of adhesion molecules and enhancement of the Nlrp3-dependent inflammatory cascade.	3D clinostat SCCS	[40] [39] [38] [41]
Treg lymphocytes	Reduced numbers	ISS	[71]
COX-2	Increased activity in K562 cells in hypergravity. Reduced activity in K562 under microgravity	Centrifugation at 22 g for 12 h	[53]
		Clinorotation at 1 g for 12 h	[53]
5-LOX	Reduced activity in K562 cells under microgravity	Clinorotation at 1 g for 12 h	[53]
	Increased activity in hypergravity	Centrifugation at 22 g for 12 h	[53]
	Increased activity in PBMCs under authentic microgravity	ISS (ROALD 10^−4^–10^−5^ g)	[42]
NAPE	Time-dependent up regulation after 48 h of microgravity aboard the ISS	ISS (RESLEM 10^−4^–10^−5^ g)	[74]
FAAH	Time-dependent down-regulation after 48 h of microgravity aboard the ISS	ISS (RESLEM 10^−4^–10^−5^ g)	[74]
AEA	Reduced levels in volunteers experiencing motion sickness	Parabolic flight	[76]
2-AG	Reduced levels in volunteers experiencing motion sickness	Parabolic flight	[76] [75]
LTB_4_	Increased synthesis	RCCS (7.2 rpm)	[54]
Anti-inflammatory cytokines	Reduced production	ISS (IMMUNO)	[71]
Pro-inflammatory cytokines	Increased production	ISS (IMMUNO)	[71]
Anti-apoptotic cytokines	Reduced production	RCCS (7.2 rpm)	[54]
Pro-apoptotic cytokines	Increased production	RCCS (7.2 rpm)	[54]

## Data Availability

Not applicable.
